# EEG-based spatial elements optimisation design method

**DOI:** 10.1007/s44223-022-00017-6

**Published:** 2022-11-21

**Authors:** Zihuan Zhang, Zao Li, Zhe Guo

**Affiliations:** 1 Faculty of Architecture and Arts, Hefei University of Technology, Hefei, China; 2 Anhui Jianzhu University, Hefei, China

**Keywords:** EEG, Spatial optimisation, Real-time interaction, Multi-objective genetic algo-rithm, TGAM module

## Abstract

In the field of digital design, a recent hot topic is the study of the interaction between spatial environment design and human factors. Electroencephalogram (EEG) and eye tracking can be used as quantitative analysis methods for architectural space evaluation; however, conclusions from existing studies on improving the quality of spatial environments based on human factors tend to remain qualitative. In order to realise the quantitative optimisation design of spatial elements from human physiological data, this research used the digital space optimisation method and perceptual evaluation research. In this way, it established an optimisation method for built space elements in real-time using human psychological indicators. Firstly, this method used the specific indicators of the Meditation value and Attention value in the human EEG signal, taking the ThinkGear AM (TGAM) module as the optimisation objective, the architectural space colour and the window size as the optimisation object, and the multi-objective genetic algorithm as the optimisation tool. Secondly, this research combined virtual reality scenarios and parametric linkage models to realise this optimisation method to establish a tool platform and workflow. Thirdly, this study took the optimisation of a typical living space as an example and recruited 50 volunteers to participate in an optimisation experiment. The results indicated that with the iterative optimisation of the multi-objective genetic algorithm, the specific EEG index decreases significantly and the standard deviation of the in-dex fluctuates and decreases during the iterative process, which further indicates that the optimisation method established in this study with the specific EEG index as the optimisation objective is effective and feasible. In addition, this study laid the foundation for more EEG indicators and more complex spatial element opti-misation research in the future.

## Introduction

### Backgroud

With the development of the world economy, including culture, science and technology, humanity’s demand for space in life and production is increasing. In this process, people will face varying requirements for different populations and different emergencies. The sudden spread of COVID-19 in 2019 not only challenged human physiology but also had a great impact on human psychology. The Secretary-General of the United Nations, Guterres, said that COVID-19 not only attacked our bodies but also increased mental distress, which seriously affected the mental health and well-being of the whole of society. The long-term isolation caused extensive socio-economic losses during the epidemic, and the loss of income and livelihoods is causing social and psychological distress. On the one hand, people with mental illness were more likely to be infected with neocoronavirus (Alshammari & Alshammari, 2021) while, on the other hand, mass isolation measures and mental health factors such as anxiety, depression and stress increased cases of infection with the neocoronavirus (Yj et al., 2021).

Besides these recent issues, the design of the spatial environment needs to meet human physiological and psychological needs, especially for children, the elderly and pregnant women in vulnerable groups. People’s satisfaction with various elements of architecture and their spatial environment is particularly important to their mental health. Architectural space is closely related to people’s psychological space. The colour of an enclosure, the light in the environment, the outline of objects and even the style of buildings will affect people’s feelings (Huang & Xu, 2009) (Li, Sun, et al., 2021, b).

### Previous studies

From previous studies on the correlation between Electroencephalogram (EEG^1^) signals and psychological quantities such as human emotion and psychological stress, it is theoretically feasible to judge people’s emotional characteristics (Msa et al., 2021) and quantify people’s degree of relaxation and stress through EEG signals (Katmah et al., 2021) (Devi et al., 2020). In clinical studies on different mental states and physical health, some relevant studies have shown that adding therapeutic meditation to standard weight loss treatment can significantly reduce stress and produce positive changes in the dietary behaviour of overweight and obese women (Sampaio et al., 2021). Another study found that mindfulness meditation can benefit people with epilepsy when practising mindfulness meditation (Delorme et al., 2020). In summation, the mental state represented by specific EEG characteristics can have a significant positive impact on human psychology and physiology (Fell et al., 2010). Enhancing people’s meditation training and enhancing the meditation value corresponding to the ThinkGear AM (TGAM) EEG module can reduce people’s stress and anxiety to a certain extent (Amha et al., 2021) (Kanchibhotla et al., 2021).

In the field of perceptual engineering, research into the human perceptual cognition of space is developing. Some studies have collected physiological data such as from human eye-tracking and EEG experiments (Li & Munemoto, 2010), combined with Semantic Differential (SD) psychological evaluation (Li, Sun, et al., 2021, b) to obtain a series of links between spatial elements (waterscape area, object contour, illumination (Lu et al., 2020), colour (Li et al., 2021), etc.) and human physiological data, going on to obtained relevant environmental improvement suggestions. To sum up, EEG monitoring methods have great research potential in the fields of architecture (Shan et al., 2018), landscape (Li et al., 2021), urban design (Prabhakar & Rajaguru, 2016) and art (Macruz et al., 2022). These studies also fully illustrate the feasibility of applying the EEG method to building evaluation and space design.

Through the above research, it can be found that human beings will produce different brain wave states under different spatial atmospheres or elements. Also, EEG signals in different states can correspond to different psychological states (Kandel et al., 2013) to produce different medical effects in long-term clinical observations. It can be concluded that it is theoretically feasible to optimise the colour, material, space volume, door and window size of architectural spaces via taking the specific EEG state as the optimisation goal, thus producing a spatial environment that can promote human mental health.

Compared with the field of building physics (Lakhdari et al., 2021) (Suga et al., 2010), building structures (Li, 2012) and other areas (Aljalal et al., 2020) (Shen et al., 2021), the relationship between human physiological data and spatial elements established in the above research does not seem to be accurate on a quantitative level, with the overall optimisation studies preferring qualitative suggestions. Huang Weixin of Tsinghua University used the IGA (interactive genetic algorithm) system to make subjects select a site, calculate and iterate according to the selection, and finally get a better parameter value of indoor space elements, thus opening up a precedent for the optimisation of perceptual engineering iterative algorithms. In their research, human perceptual evaluation is used as a reference for interactive optimisation, which provides some inspiration for this study (Huang & Xu, 2009).

### Research aim

**Fig. 1:**
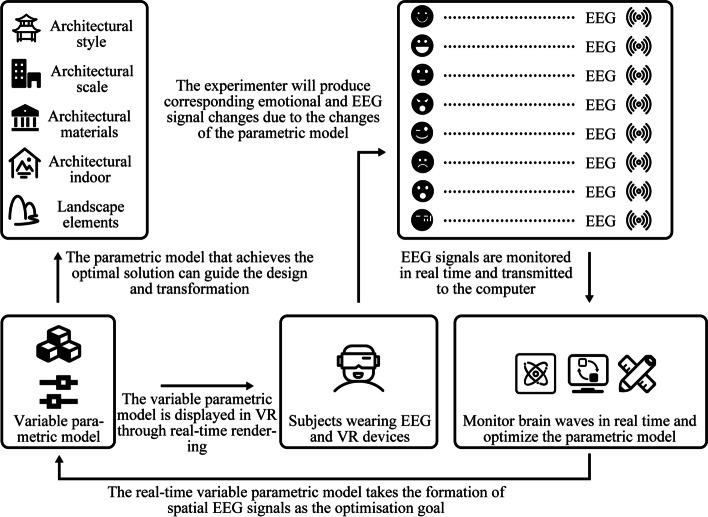
EEG-based spatial optimisation method

This study aims to create an effective architectural design optimization method, which is a quantitative optimization method of architectural space elements based on the physiological data of human emotion and psychological elements. Figure 1 shows the goal vision of this approach.

This method is used to form a closed-loop optimisation participated in by people. People do not need to make active judgments in the optimisation process. During optimisation, the subject observes the virtual reality scene and the EEG signal is monitored in real-time by the TGAM EEG module. The optimisation algorithm changes the parametric linkage model in real-time via referring to the EEG value, changing the virtual reality scene observed by the subject. The process is iterated continuously in the black box until the optimisation result tends to be stable (in theory, the optimisation of the genetic algorithm can go on indefinitely). In this study, the specific indexes of meditation value and attention value in the human EEG signal are analysed with the head ring of the TGAM module as the optimisation goal, the colour of building space and window opening size as the optimisation object and the multi-objective genetic algorithm as the optimisation tool. Combined with the virtual reality scene and parametric linkage model, the tool platform and workflow used to realise this method is established. Thus, the research aims of this study consist of the following three points:

- Develop a tool platform for real-time interactive spatial element optimisation based on EEG signals. The tool platform is composed of a hardware system and a software system.
- Establish the workflow of real-time interactive spatial element optimisation based on EEG signals.
- Carry out multiple groups of optimisation experiments of real-time interactive spatial element optimisation methods based on EEG signals, complete the evaluation of the optimisation methods and propose improvement goals through quantitative analysis of experimental data and optimisation results.

## Method

### Tools platform

In the experiment, this research involves electrical signal communication, real-time rendering of virtual reality scenes and optimisation and linkage of the parametric model, meaning the workflow is relatively complex. The required work platform is divided into a hardware platform and a software platform.

On the hardware platform, this study assembled a single-electrode ear-clip brainwave head-ring through the TGAM EEG module (Yin et al., 2020); this can monitor human brain waves in real-time, α Wave, β Wave, γ Wave, etc., and calculate people’s meditation value and attention value through the black box (meditation here represents people’s sense of calm and pleasure, which is a relaxing EEG feature, and attention here represents concentration value, which is an EEG feature generated when people pay attention and tension in the brain). As an open-source and low-cost EEG module, the TGAM (ThinkGear AM) module has been widely used in scientific research (Jadhav & Momin, 2018) (Peterson et al., 2020). The author believes that optimisation based on psychological feeling in the design stage of the construction field in the future will meet a market demand. The development of the tool platform needs to be considered in combination with many factors, such as tool price, ease of use, resistance to the influence of complex external environment, user comfort and so on.

In this study, the TGAM EEG module will continuously send human EEG signals through Bluetooth in real-time during the experiment. The EEG signals are pre-processed through the Arduino development board and the processed meditation value and attention value are sent to the computer’s serial port. The computer is used to run the optimisation program and transmit the changing parametric model to Oculus Rift S virtual reality glasses through real-time rendering. Other hardware devices (in addition to the devices used in this study) have the potential to be adapted to implement this method; for example, EEG acquisition devices include the BrainLink Pro, which uses the TGAM chip, and the DIY Neurotechnologist’s Starter Kit, which uses the Open BCI chip. The choice of VR glasses should combine their compatibility with real-time rendering software. Both Oculus rift s and HTC Vive have good compatibility. In addition, the computer hardware configuration recommended is a high performance computer with an independent graphics card.

**Fig. 2:**
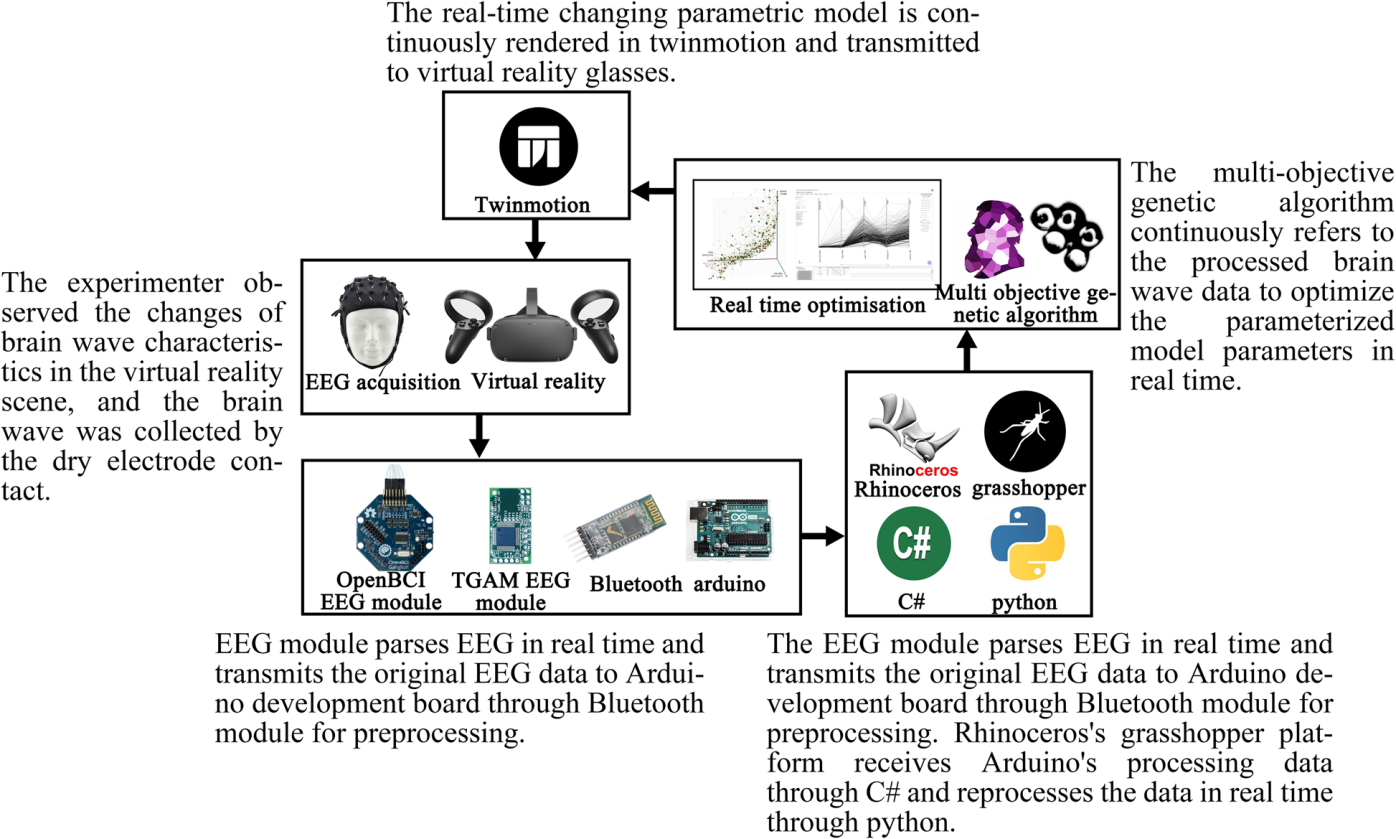
EEG-based spatial optimisation tool platform workflow

On the software platform, the preprocessing of the uploading program of the Arduino development board is completed by writing C language in Arduino ide. Its purpose is to capture and process the original EEG data sent via the TGAM EEG module, convert hexadecimal into binary language and input it into the computer serial port. Because Grasshopper can cooperate with multiple programming languages and is suitable for building parametric models, this study uses the Grasshopper platform for the optimisation algorithm. On this platform, this research uses the data read from the serial port as the reference of the optimisation algorithm in real-time, establishes the standard bedroom unit with variable colour and window hole size, links it to the real-time rendering software based on twinmotion platform through the program, and transmits the virtual reality scene to the Oculus Rift S virtual reality glasses through twinmotion. In this study, a wallacei multi-objective genetic algorithm based on the Grasshopper platform is used as the main optimisation algorithm to optimise the colour and window opening size of typical bedroom units with real-time reference to the optimisation objectives. Figure 2 shows the workflow of the optimisation tool platform.

### Workflow

This section includes the establishment of the initialisation virtual scene, the establishment of a variable library and the design of the basic experimental process. The design of the basic flow of the experiment includes the methods of establishing the key steps of this research, such as the real-time reading and processing of EEG data on the Grasshopper platform, the real-time rendering linkage of the parametric model and virtual reality scene and the adaptation of a black-box optimisation algorithm on the Grasshopper platform.

#### Establishment of initial virtual reality scene

The real-time interactive optimisation experiment has high requirements for the linkage between software platforms. In this study, a bedroom model with a bay of four meters, a depth of five meters and a height of three meters is established on the Grasshopper platform. The window is located on the south side, and the initial window size is to scale the south wall shape on its plane to 0.5 times the original shape. The initial indoor colour of this bedroom model is white (all three RGB values are 255). The interior model does not contain other objects and the colour and material characteristics of the floor, wall and ceiling are the same.

#### Establishment of variable database and optimisation objectives

As mentioned above, the initialisation scene is the optimisation subject of this study. The typical bedroom model of this scene will be observed through indoor observation in this study. The subject contains five optimisation variables; namely, the variable parameters controlled by the program in the optimisation process.

In previous studies, it has been found that light and colour have a great impact on people’s psychological feelings in a scene (Li Z 2021a). Therefore, in this study, the window hole size and indoor colour that control the amount of light will be optimised, where the variables controlling the window hole size are X-axis zoom and Y-axis zoom, and X-axis zoom controls the width of the window hole. The Y-axis scaling amount controls the height of the window opening. When the X and Y scaling amounts are 1, the window opening is as large as the entire wall. When the scaling amount is 0, the window opening disappears. The colour variables in the control room are R, G and B. See the following table (Table 1) for specific variable elements, span and accuracy of variables.

**Table 1:** Construction of the window opening and colour optimisation variables Library

Optimised object	Optimisation object	Variable span	Number of variables
Window opening	X-axis zoom	0.10 — 0.90	90
	Y-axis zoom	0.10 — 0.90	90
Interior colour	R	0 — 255	256
	G	0 — 255	256
	B	0 — 255	256

In this study, there are two optimisation objectives in the operation of the multi-objective genetic algorithm, namely Attention and Meditation. Attention represents the degree of stress and concentration, the smaller the better, while the value of Meditation represents the degree of relaxation and pleasure, the larger the better. However, in this experiment, due to the characteristics of genetic algorithms, the optimisation objective was approached to the minimum value in the operation, so the negative value of Meditation was used in the calculation process. The formula is as follows, where Average_Meditation represents the average value of Meditation calculated when the subject observes each scene, while Meditation’ represents the value calculated by the genetic algorithm after negative processing. The average value of Attention only needs to be calculated to generate Average_Attention, which is directly inputted into the genetic algorithm for calculation. See below for the specific average calculation method.

1 $${Meditation}^{'}=- Average\_ Meditation$$

#### Arduino board program uploading method

**Fig. 3:**
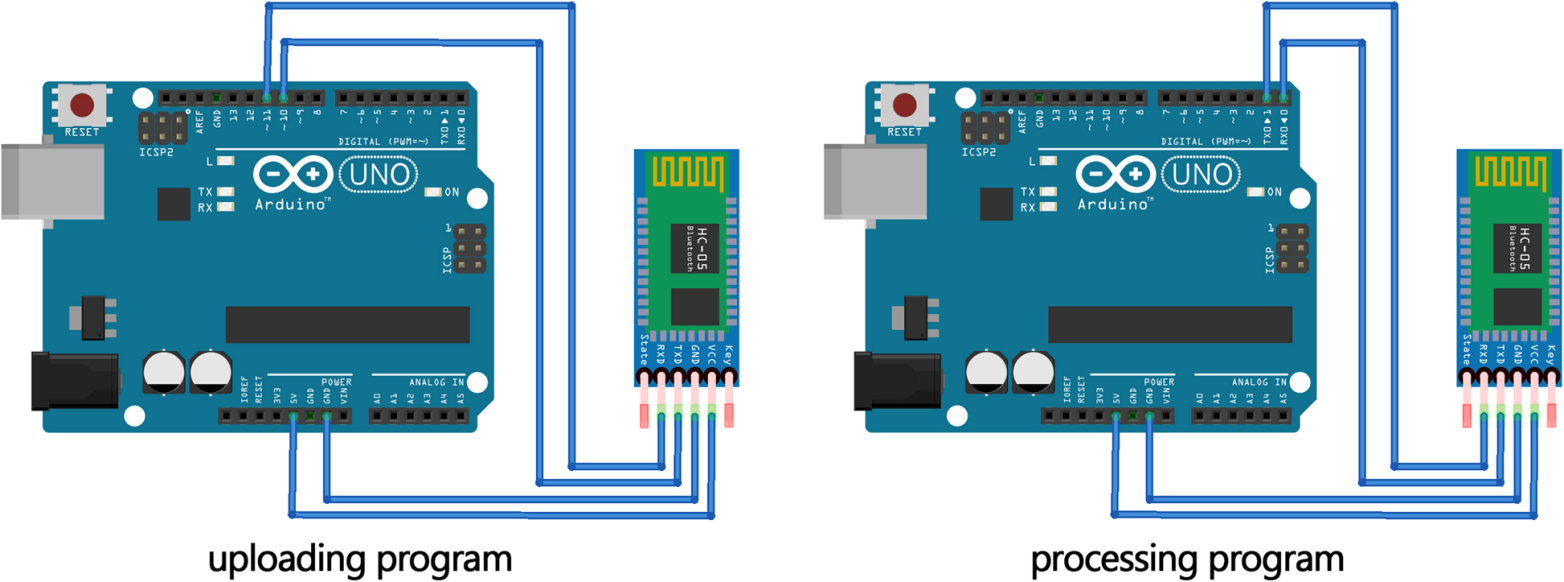
Arduino board program uploading method

The EEG signal based on the TGAM EEG module in this study needs to be pre-processed in the Arduino development board so the Arduino board can read the EEG data in real-time and capture the attention value and meditation value in the TGAM module before transmitting them to the Grasshopper platform through the serial port. Before that, it needs to upload the program to the Arduino board. The EEG mode of receiving the TGAM EEG module on the Arduino board is Bluetooth reception. The pin connection method during program uploading is Arduino 5 V – VCC; Arduino GND – GND； Arduino Pin10 – TXD； Arduino Pin11 – RXD. After uploading, the TXD and RXD are connected to Pin0 and Pin1, respectively (Fig. 3).

#### Linkage method of the parametric model and real-time rendering of the virtual reality scene

The parametric model in this study needs real-time linkage with virtual reality glasses. On the software platform, the real-time rendering software based on Twinmotion supports the real-time rendering of virtual reality glasses, but the Twinmotion model needs to come from the modelling software. Through programming, this study solves the problem that changing the parameters of model variables can lead to the real-time change of the virtual reality scene through the real-time linkage between the Grasshopper platform and Twinmotion. The key code is realised via calling “scriptcontext. Doc. Objects. Add” and “Datasmithdirelinksync” instructions. Its function is to add objects in Grasshopper to Rhino and refresh the linkage between Rhino and Twinmotion.

### Adaptation of the optimisation algorithm

There are many excellent optimisation algorithms on the Grasshopper platform, such as Galopalos, Octopus, Wallacei etc. Wallacei^2^, based on the NSGA-II algorithm, is selected in this study. Wallacei has strong advantages in the selection and optimisation of results, as well as results analysis after optimisation, and takes the k-means method as the clustering algorithm which is conducive to the analysis of a large number of sample data.

The TGAM EEG module collects the EEG signals of the subjects through the electrodes of the prefrontal lobe and sends the subjects’ current meditation value and attention value every second through the module calculation. Both values range from 0 to 100. This study hopes to obtain the space that indices a higher meditation value and lower attention value through an optimisation calculation to reduce people’s sense of tension and anxiety in the environment and increase their sense of relaxation. During the experiment, to ensure that the experimenter can produce more reasonable EEG feedback corresponding to the scene during observations, each scene is stopped in front of the experimenter for 5 s (Li & Munemoto, 2010) (Sun & Li, 2021) (Cui et al., 2021); the EEG values of the first and last seconds are discarded and the average value of the EEG in the middle section is taken as the reference value for the genetic algorithm (objective). The specific formula is as follows. In the following formula, t_0_ and t_1_ represent the moment 1 s after the beginning and 1 s before the end of each scene change, respectively.

2 $$Average\_ Meditation=\frac{\int_{t_0}^{t_1} Meditation\bullet dt}{t_1-{t}_0}$$

3 $$Average\_ Attention=\frac{\int_{t_0}^{t_1} Attention\bullet dt}{t_1-{t}_0}$$

**Fig. 4:**
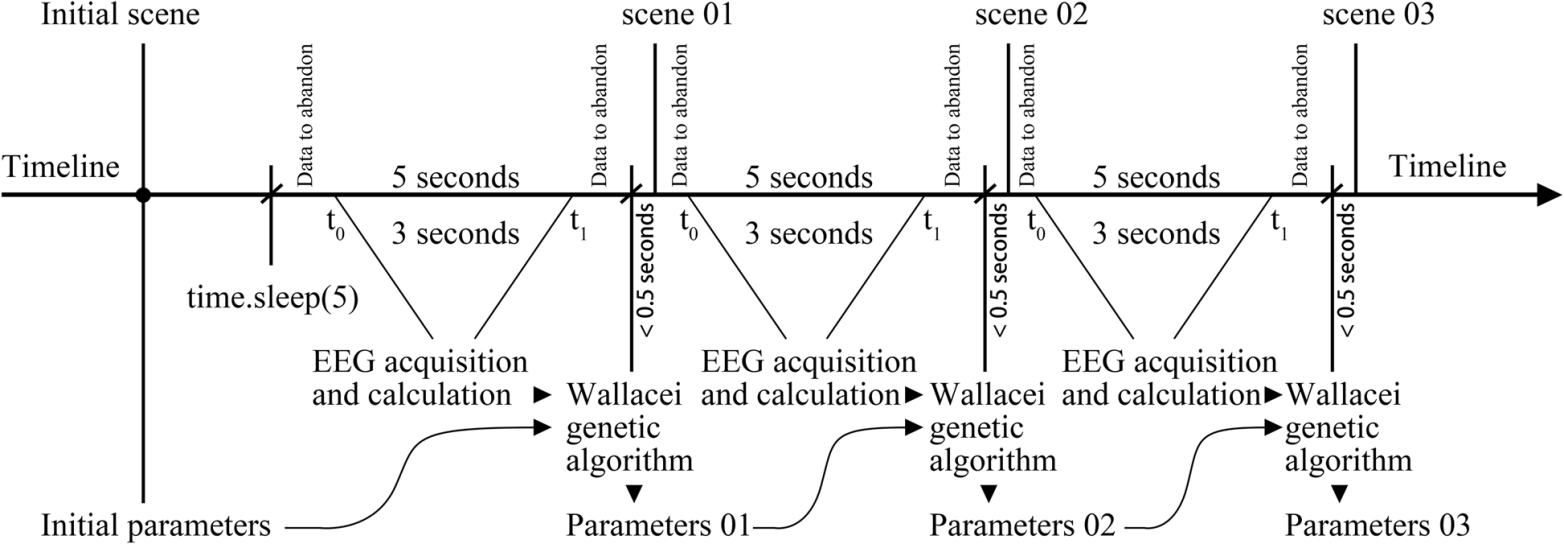
Time axis diagram

Based on the above, a group of EEG processed data can be generated every 5 s during the experiment as the optimisation reference of the optimisation algorithm, but it is impossible to make the optimisation algorithm wait for 5 s. In previous research using genetic algorithms, machines participate in the simulation and calculation, so the general setting of the algorithm is to maximise the performance – to speed up the optimisation speed. In this research, human observation participation is generated so the optimisation algorithm needs to wait in the process of optimisation calculation. Here, the problem is solved by the “time. Sleep (5)” algorithm. Figure 4 shows the time relationship in the experiment in detail.

It is worth noting here that “time. Sleep (5)” in ghpython will cause all calculations in the whole grasshopper platform to stop, while the meditation value and attention value in the serial port are read by C# language and will not stop reading. Within the time when other calculations stop for 5 s, the meditation value and attention value will accumulate 5 s of data. At the end of 5 s, it is inputted as a list to calculate the average value (1) (2) which is transmitted to the genetic algorithm as a reference for calculation. The time of this process was proven to be within 0.5 seconds after many tests, which is lower than the time of the previous second discarded in the average calculation, and will not be superimposed with time so it will not affect the accuracy of the optimisation algorithm.

### Experimental preparation

To test the applicability of the optimisation method in this study, 50 volunteers of different ages were recruited for the optimisation experiment. Of the 50 volunteers, 52% were male and 48% female, with 4% children, 76% youth, 16% middle-aged, and 4% elderly. This study focuses more on the applicability, logic of the method, and effectiveness of optimisation. This section will discuss the experimental environment preparation, experimental process, experimental results and analyses.

**Fig. 5:**
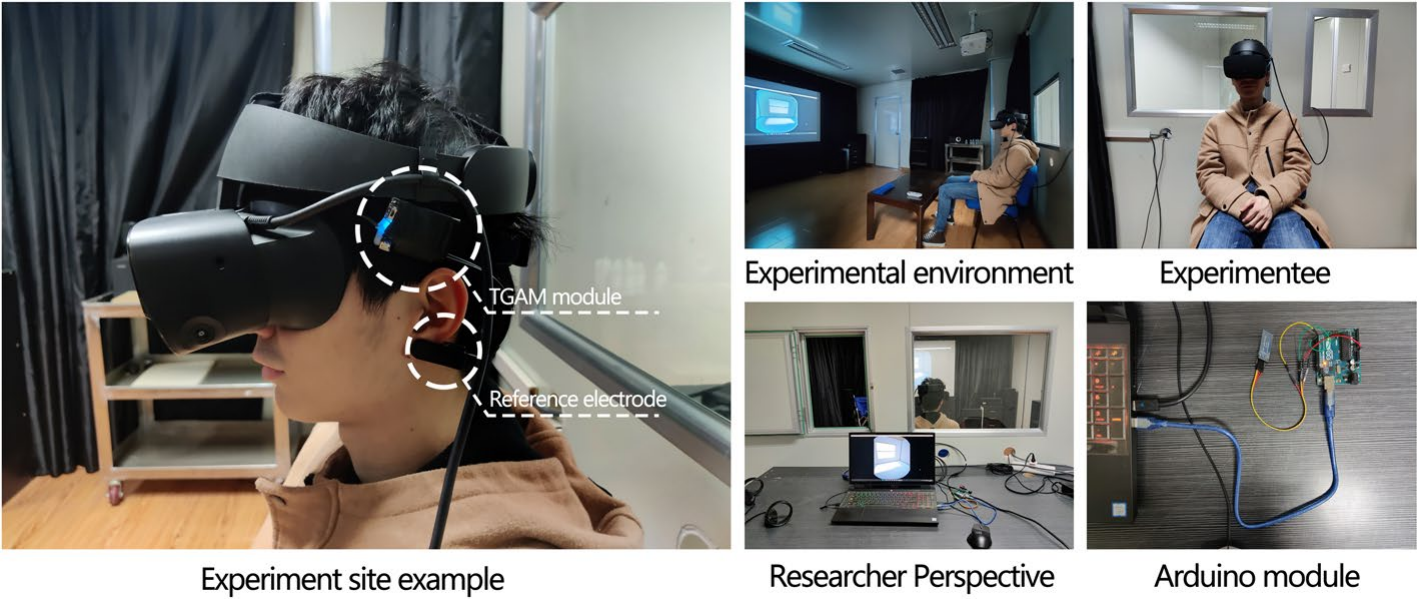
Experiment site example

As shown in Fig. 5, the experimental environment is a special EEG and Eye movement laboratory. The laboratory provides the subject with an environment with less external interference and allows the researcher to observe the experimental process. Before the experiment, the subjects wear EEG equipment first and then wear VR glasses. After the EEG runs stably, the experiment can be started. The genetic algorithm in the experiment is initially set to iterate (Generation Count) 20 times, with 10 biomass (Generation Size) per generation. The experimental time is estimated by the software to be about 18 minutes and 31 seconds.

In the early stage of this study, some pre-experiments have been conducted to explore the appropriate number of iterations, including five iterations, 10 iterations, 20 iterations and 30 iterations. In these experiments, it was found that the iterative optimisation gradually reaches the optimal result at about the 15th generation and gradually becomes stable from the 15th generation to the 20th generation. According to the post-test interview, after 25 generations of iteration, some subjects would be tired due to the long experiment time. Therefore, taking into account the physical and mental health of the subjects and the effect of the optimisation experiment, this study selected 20 iterations in the formal experiment.

## Experimental results and quantitative analysis

### Analysis of optimisation effect

**Fig. 6:**
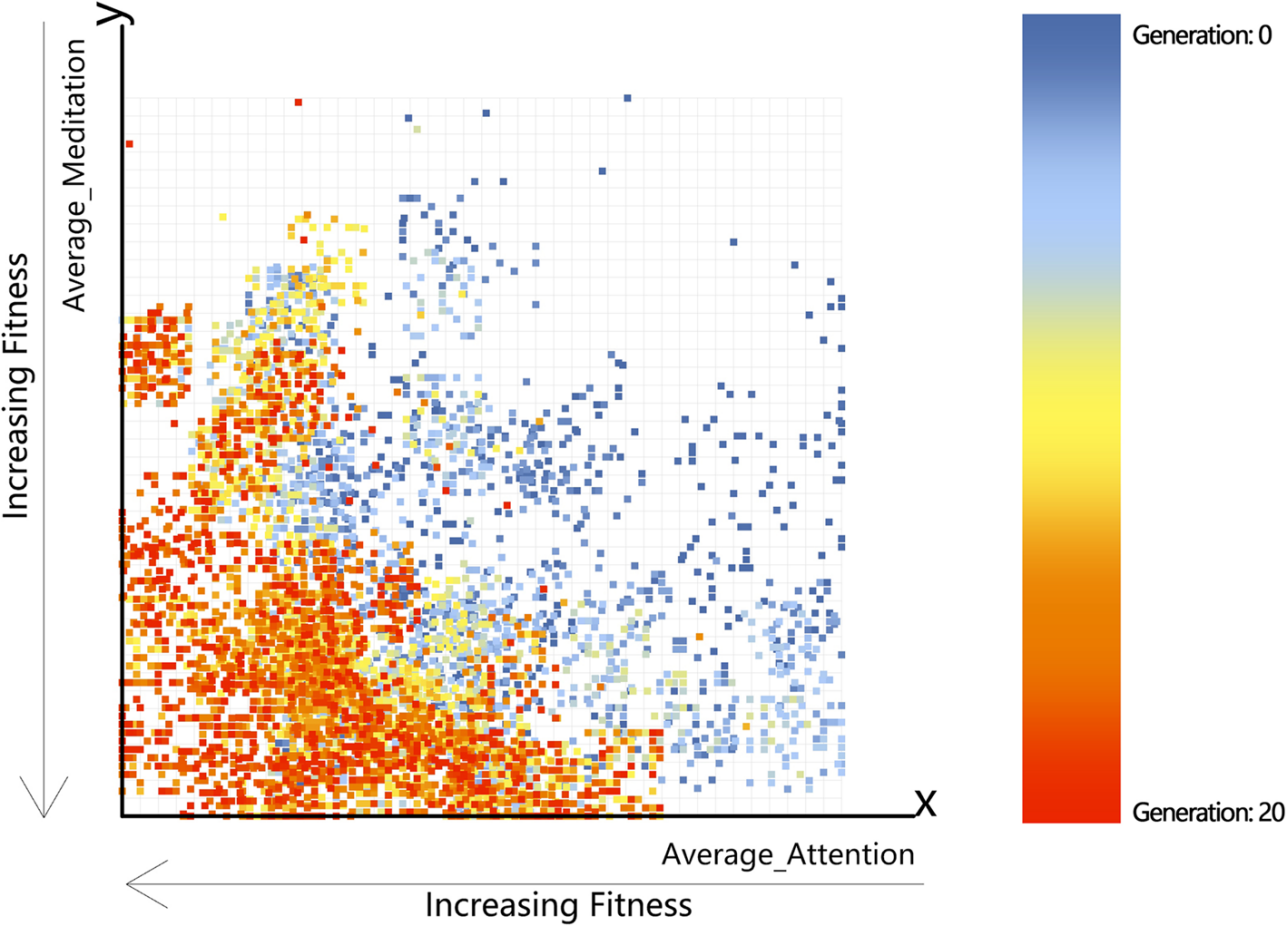
Objective Space

Figure 6 shows the optimisation target distribution diagram of 10,000 optimisation target data in the optimisation experiment of 50 volunteers.

After completing the optimisation, Wallacei combined with its analysis system can sort the optimisation results according to their advantages and disadvantages, indexing the “generation” and “index” generated by samples in the genetic algorithm optimisation, where “generation” is abbreviated as Gen and “index” is abbreviated as Ind. “Generation” represents the number of generations in which the sample is located and “index” represents the index of ten biomass in each generation. Through optimisation, even one of the subjects has produced better results with attention as low as 2.5 and meditation as high as 98.5. Indexing the result obtains (Gen: 19 | ind: 3); that is, the 4th individual of the 20th generation (the index in the computer starts from 0 by default).

**Fig. 7:**
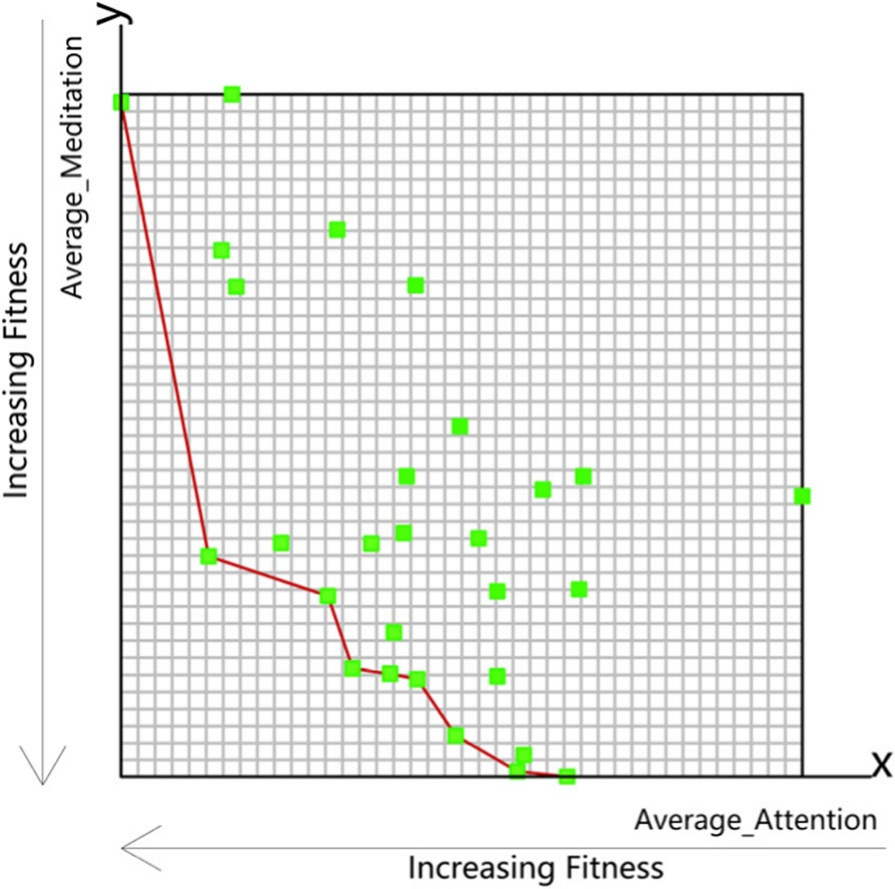
Objective Space of one Subject

The Objective Space (OS) re-maps the optimisation target value of the analogue output and specifies a different axis for each target. Average_Attention and Average_Meditation are displayed on the X and Y axes, respectively. As shown in Fig. 6, all samples from the subjects are in the form of a scatter diagram, a warmer colour representing an increasing number of iterations and a colder colour indicating fewer iterations. When the sample points are close to the origin, it can be said that the optimisation effect is good. It can also be concluded from the figure as the optimisation iterations, the sample is getting closer to the origin, then explain the optimisation effect is obvious. For one of the subjects, as shown in Fig. 7 where the green dots in the figure represent the non-dominant Paredo optimum of each generation, optimisation was formed by the 20th generation with a Wallacei Non-dominated Pareto polyline (red line). According to the red line, there were nine Non-dominated Pareto optimal solutions of the 20th generation result.

To sum up, nine excellent optimisation results can be obtained from the subject using the OS analysis method. Table 2 shows example optimisation results from one subject. It can be seen from this table that the best optimisation results from the subject are achieved at the 20th generation after multiple iterations of optimisation. The variables in the optimisation results show some trends. The X_Scale and Y_Scale values are mostly around 0.5, the R values are relatively small with some deviation between samples and the G and B values are relatively large. The difference between the Meditation value and the Attention value was more than 40, and the gap was obvious. A more specific analysis of all the subjects is presented in Section 3.2.

**Table 2:**
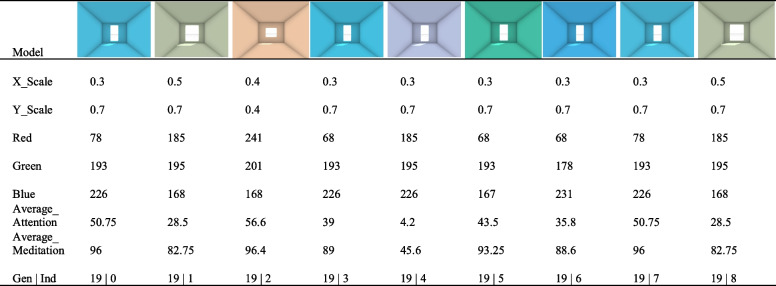
Example of optimisation results

**Fig. 8:**
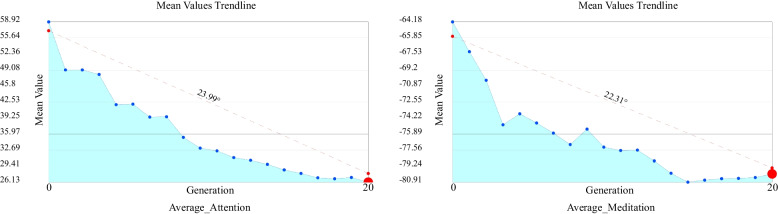
Mean value trendline of Average_Attention and Average_Meditation values

From the entire optimisation process, referring to the generation average value trend chart (MV) of the optimisation target shown in Fig. 8, it can be seen that with the optimisation iteration, the Average_ Attention value decreased obviously during the optimisation process while the Average_Meditation calculated by the negative value showed a significant decrease in optimisation; that is, the positive value of this value increased significantly. The larger red dot in Fig. 8 represents the average value of the optimisation target generation of the results of the 20th generation. It can be seen that the optimisation of window opening size and indoor colour based on the EEG signal in this study is effective.

**Fig. 9:**
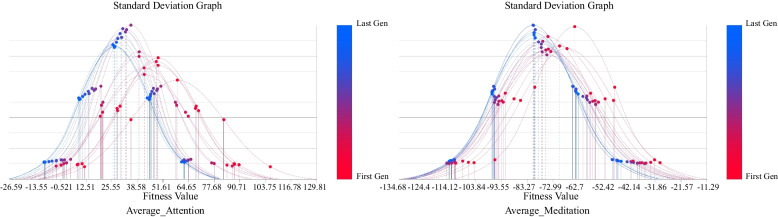
Standard deviation graph of Average_Attention and Average_Meditation values

**Fig. 10:**
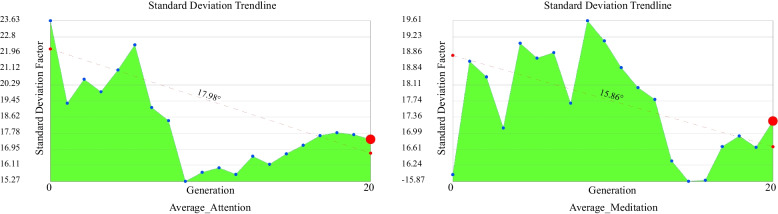
Standard deviation trendline of Average_Attention and Average_Meditation values

The duration of this experiment is 18 min and 31 s. There is a possibility of the subjects becoming sleepy during the experiment, resulting in the homogenisation of the subjects’ brain waves, that is, they no longer produce effective feedback with changes to the scene which leads to inaccuracy in later optimisation readings. To eliminate the possibility of this situation, this study further analyzes the experimental results and data. The analysis focuses on multiple individual standard deviations of EEG feedback in different iterative processes. If the standard deviation decreases significantly over time, this shows the subject’s EEG feedback with scene changes decreases. The standard deviation represents the distribution of a set of mean values. A low standard deviation coefficient indicates that most values are clustered around the average (with small changes in the population), while a high standard deviation coefficient indicates that the values are distributed further away from the average (with large changes in the population). The purpose of the standard deviation graph (SDG) is to show and analyse the variation and convergence level of each generation in the population and whether each generation becomes a better fit during the simulation process. An increased change is represented by a “flat” curve while an increased convergence is represented by a “narrow” curve and moving the curve to the left indicates better average performance (Fig. 9). The colours of these generations range from red (the first generation) to blue (the last generation). The standard deviation (SD) (4) chart calculates the judgment value that represents the degree of dispersion of each generation in the population and plots each generation as three standard deviations on both sides of the mean. The red dots in the Fig. 10 highlight the values corresponding to the last generation of iterative optimisation.

4 $$\sigma =\sqrt{\frac{1}{N}\sum\nolimits_{i=1}^n{\left({x}_i-\mu \right)}^2}$$

From the analysis, it can be concluded that during the experiment, the standard deviation of EEG values for attention and meditation, the optimisation target, fluctuated greatly as the experiment progressed and there was no obvious downward trend but even increases in some stages. To sum up, the analysis shows that the subjects have effective feedback to changes in the virtual reality scene while participating in the optimisation experiment. This can be explained to a certain extent as this experiment does not induce sleepiness in the subjects during the experiment which would lead to inaccuracy in experimental results (Fig. 10).

### Analysis of the optimisation results

RGB colour space is the most basic, the most commonly used, hardware-oriented colour space in image processing and is relatively easy to understand. However, images acquired in the natural environment are easily affected by natural illumination, occlusion and shadow; that is, they are sensitive to brightness. The three components of RGB colour space are closely related to brightness; that is, as long as the brightness changes, the three components will change accordingly but there is no more intuitive way to express this. HSL and HSV colour space are more often used in image processing, which is closer to people’s perceptual experience of colour than RGB. HSL has three main components, hue, saturation and lightness, which express the hue, vividness and shade of a colour directly and facilitate the comparison of colours.

**Fig. 11:**
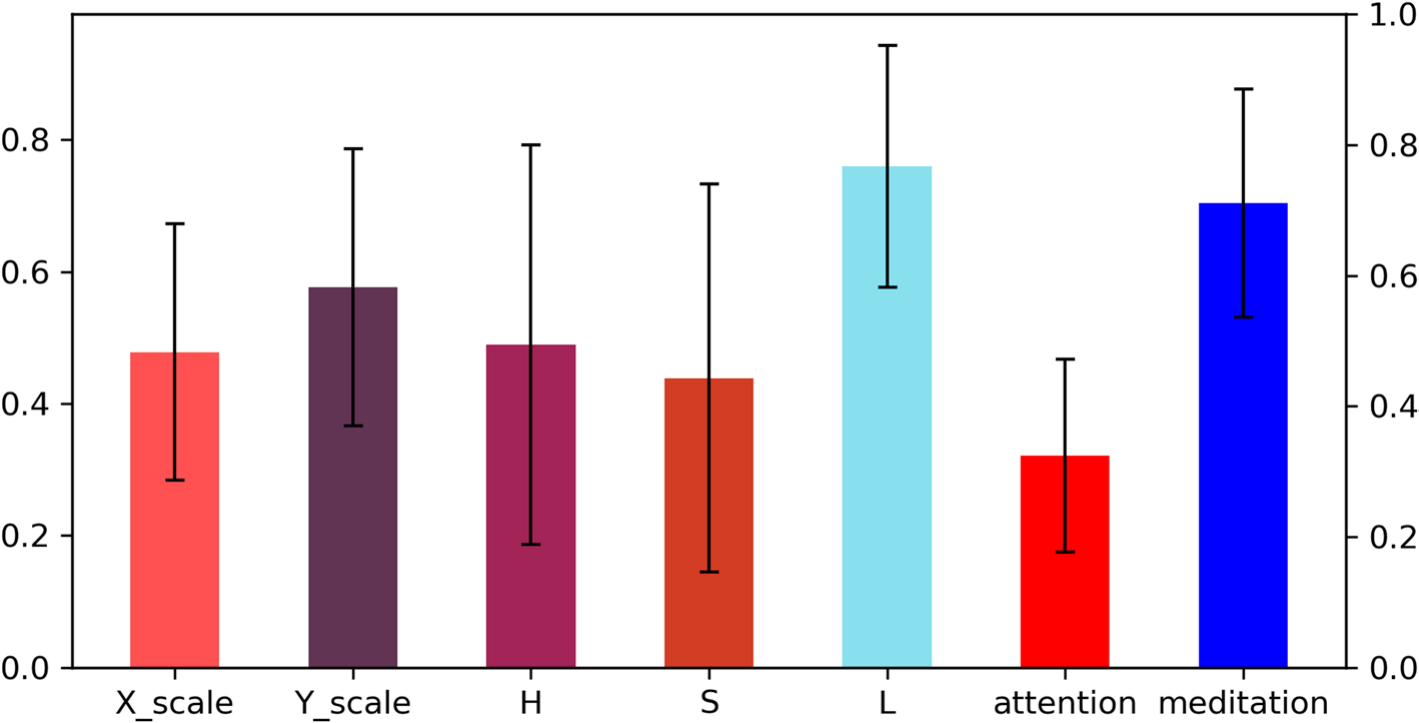
Optimisation results statistics

Generally speaking, the sensitivity of the human eye to the three colour components of red, green and blue is different, so the uniformity of the RGB colour space is poor. In order to make the genetic algorithm and colour index more suitable for human visual participation, this study introduced the HSL colour space model in the study of the optimisation results and again recruited 20 healthy volunteers aged 23 to 28 years to participate in the optimisation experiment. A total of 102 optimisation results were produced in the experiment and 90% of the subjects were satisfied with the optimisation results through on-site interviews. Figure 11 shows the average value and standard deviation of each indicator of the optimisation result, where the attention value and the meditation value are standardised values.

**Fig. 12:**
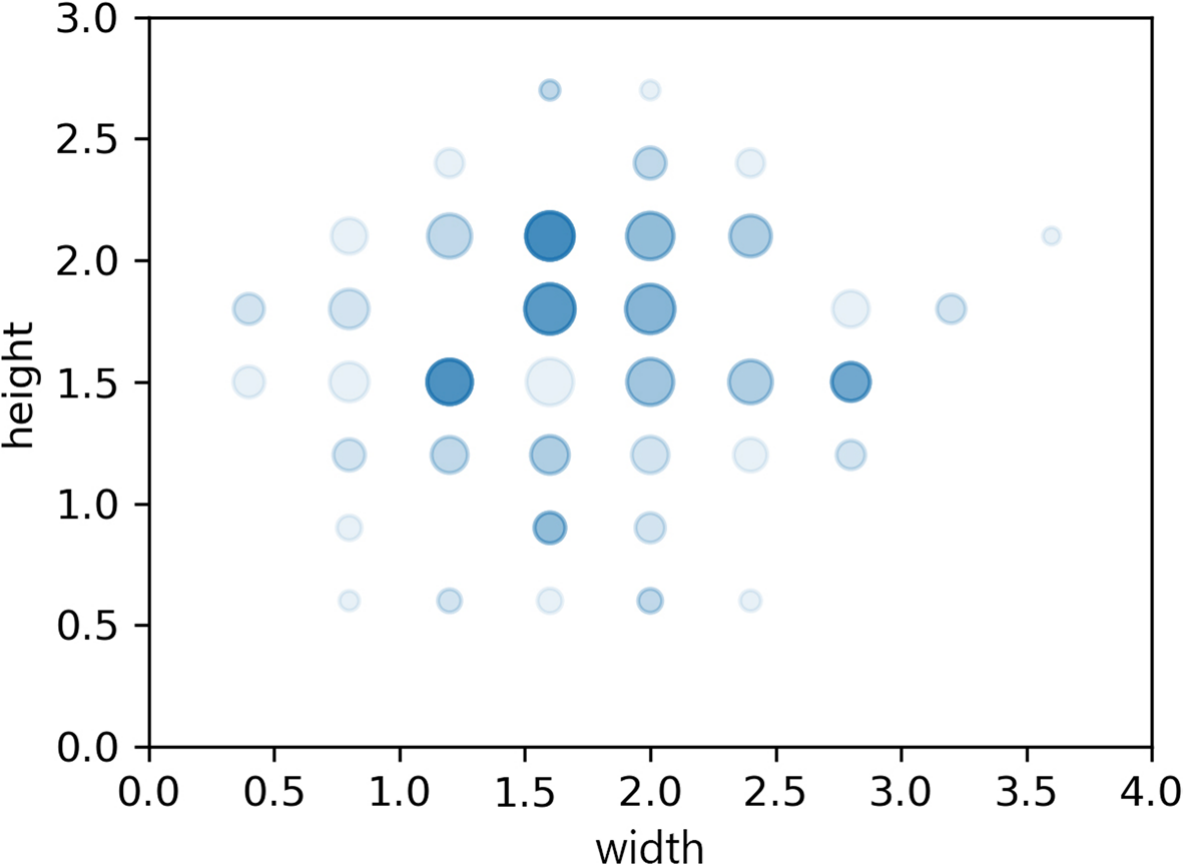
Window size scatter distribution in optimisation results

According to the statistical chart of the optimisation results, the standard deviation of the attention value and the meditation value is small. While the attention value is small as a whole, mainly floating around 0.3, the meditation value is large as a whole, mainly floating around 0.7, indicating that the optimisation result is good. The X-axis scaling of the window hole size is mainly 0.4 to 0.6, the Y-axis scaling is mainly 0.5 to 0.7, and the Y-axis scaling is slightly larger than the X-axis scaling. As shown in Fig. 12, the figure represents the scatter distribution of the window size in the optimisation result. The X-axis and Y-axis of the figure are in meters, which is the result of scaling multiplied by the actual size of the wall. The larger the number of overlapping scatter points in the figure, the larger the scatter size and the larger the colour concentration. It can be seen from the figure that most of the optimisation results are distributed in the upper left area in the middle of the figure, and the height of the window hole is slightly larger than the width.

Compared with the H value representing hue and S value representing the saturation, the L value has a smaller standard deviation and the overall value of L value is large and mainly fluctuates around 0.8, while S value is small. This indicates that indoor colours with high brightness and low saturation are more conducive to people in a state of meditation. The distribution of the H values is wide, which shows that the hue index has individual differences in the experimental optimisation results.

**Fig. 13:**
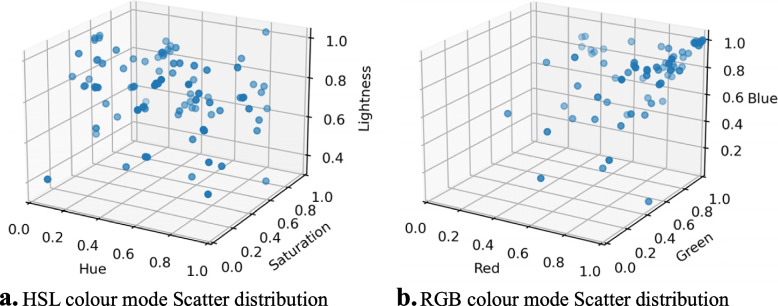
Colour scatter distribution in optimisation results. **a** HSL colour mode Scatter distribution. **b** RGB colour mode Scatter distribution

Figure 13 shows the scatter plot of colour information of the optimised experimental results, where the left figure is the scatter plot of the HSL colour mode and the right figure is the scatter plot of the HSL colour information converted into an RGB value, where RGB value is the normalised value. The RGB values of colours obtained from the RGB scatter distribution map are mostly clustered on the side with larger values.

After the experiment, a study on the subjects’ satisfaction with the outcome of the experimental optimisation was carried out with a questionnaire and interviews. A questionnaire of all the optimisation result shows the subjects’ results, such as in the above Table 2, each of which showed subjects were “very satisfied”, “satisfied”, “general”, “not satisfied”, “very dissatisfied”. The results of the questionnaire were statistically analysed, as shown in Table 3 below, with 19 out of 20 subjects indicating that the optimisation results contained satisfactory results.

**Table 3:** Statistical table of post-test interview results

	very satisfied	satisfied	general	not satisfied	very dissatisfied
amount	13	65	18	4	2
percentage	12.75%	63.73%	17.65%	3.92%	1.96%

## Conclusion

In this paper, it was found that it is feasible to take human-specific EEG signals as the optimisation goal of optimising building space elements. In the experiment taking the size of the window opening and the colour of the indoor space as an example, the optimisation results were selected according to the Pareto optimal method. The optimisation effect of the genetic algorithm with the optimisation objectives of meditation value and attention value is obvious. Furthermore, according to the trend of standard deviation, the possibility that the subjects’ sleepiness affected the experimental results can be excluded.

In the above optimisation research based on the TGAM EEG module, experiments show that in typical living units, the size of the window opening is usually 0.4 to 0.6 of the X-axis scaling of the wall where it is located, the Y-axis scaling is 0.5 to 0.7 and the indoor colour is high brightness with low saturation, which is more conducive to people in a meditative state. This study will use more accurate EEG equipment in further research to further analyze the optimisation results and try to analyze the characteristics and scope of the optimisation results through clustering. Since this study is still in the initial stage of exploration, only “window size” and “interior colour”, which have obvious influences on EEG, are selected as the research objects. At present, this study has formed a certain patent technology (Zhang et al., n.d.) and plans to try to apply this technology in future research on the optimisation of actual projects, at the same time ensuring that the psychological needs and the traditional style and features of huizhou residential window inform research, as well as the old village renovation project of the ceiling height, humanly scaled form, indoor colour, material texture research.

Considering the difficulty of the experiment and the influence on the subjects, this study adopts the commonly used five-second scene EEG-recording method in the design of the experiment. In the actual situation, there are certain differences between the impact of short-term scenes and long-term continuous scenes on human beings in terms of clinical mental health impact. The selection of the final optimisation results still needs to be combined with perceptual evaluation.

The closed-loop optimisation model established in this study from EEG equipment to the grasshopper platform, as well as the real-time rendering engine to VR equipment, will be used to further optimise a wider range of scenes involving building volume, architectural style, landscape elements, etc. The applicability scenario of the optimisation algorithm in further research will be expanded and the development of more possibilities of man-machine coupling design in artificial intelligence technology will be promoted.

In further research, a more accurate OpenBCI EEG module supporting 16 electrodes will be used to build the tool platform and establish a more stable and convenient connection between virtual reality and real-time optimisation. More mental states based on EEG data will be considered and more diverse EEG data will be used as a reference for optimisation. Based on this research, complex multi-objective and multi-factor real-time interactive optimisation will become possible.

## Data Availability

The datasets generated during and analyzed during the current study are not publicly available due to protection of subjects’ personal information as well as their privacy. The raw data are available from the corresponding author on reasonable request.

## Footnotes

1 Electroencephalogram (EEG) is a method of recording brain activity using electrophysiological indicators.

2 Wallacei is developed by Mohammed makki, Milad showkatbakhsh and Yutao song.
